# Insignificant QBO‐MJO Prediction Skill Relationship in the SubX and S2S Subseasonal Reforecasts

**DOI:** 10.1029/2019JD031416

**Published:** 2019-12-05

**Authors:** Hyemi Kim, Jadwiga H. Richter, Zane Martin

**Affiliations:** ^1^ School of Marine and Atmospheric Sciences Stony Brook University Stony Brook NY USA; ^2^ National Center for Atmospheric Research Boulder CO USA; ^3^ Department of Applied Physics and Applied Mathematics Columbia University New York NY USA

**Keywords:** Madden‐Julian oscillation, quasi‐biennial oscillation, prediction

## Abstract

The impact of the stratospheric quasi‐biennial oscillation (QBO) on the prediction of the tropospheric Madden‐Julian oscillation (MJO) is evaluated in reforecasts from nine models participating in subseasonal prediction projects, including the Subseasonal Experiment (SubX) and Subseasonal to Seasonal (S2S) projects. When MJO prediction skill is analyzed for December to February, MJO prediction skill is higher in the easterly phase of the QBO than the westerly phase, consistent with previous studies. However, the relationship between QBO phase and MJO prediction skill is not statistically significant for most models. This insignificant QBO‐MJO skill relationship is further confirmed by comparing two subseasonal reforecast experiments with the Community Earth System Model v1 using both a high‐top (46‐level) and low‐top (30‐level) version of the Community Atmosphere Model v5. While there are clear differences in the forecasted QBO between the two model top configurations, a negligible change is shown in the MJO prediction, indicating that the QBO in this model may not directly control the MJO prediction and supporting the insignificant QBO‐MJO skill relationship found in SubX and S2S models.

## Key Points

- The QBO‐MJO prediction skill relationship is mostly insignificant in nine subseasonal reforecasts
- The QBO‐MJO prediction skill relationship is sensitive to the choice of QBO events
- The insignificant QBO‐MJO skill relationship is supported by comparing reforecasts by a high‐top and low‐top version of CESM1

## 1 Introduction

The Madden‐Julian oscillation (MJO, Madden & Julian, 1971, 1972), an organized envelope of tropical convection with a life cycle of about 40–50 days, is a major source of global subseasonal predictability. While there have been great advances in understanding MJO predictability and its global impacts on subseasonal timescale (see reviews by Stan et al., 2017; Kim et al., 2018), study on the year‐to‐year variation of MJO predictability has received little attention. This was partly due to the lack of reforecasts over a sufficiently long period (>10 years) as well as a lack of evidence of large‐scale basic state forcing or interannual variability influencing the MJO.

Very recently however, studies have found evidence of a connection between the quasi‐biennial oscillation (QBO) and the MJO during boreal winter in observations, especially from December to February (DJF) when the QBO‐MJO relationship is the strongest (Abhik & Hendon, 2019; Hendon & Abhik, 2018; Marshall et al., 2017; Nishimoto & Yoden, 2017; Son et al., 2017; Yoo & Son, 2016; Zhang & Zhang, 2018). DJF is also the period when the MJO is typically most active. The QBO is an oscillation of the equatorial stratospheric zonal winds between easterlies and westerlies with an observed mean period of 28 months (e.g., Baldwin et al., 2001). During the easterly QBO (EQBO) phase, the observed MJO tends to better organize and propagates further eastward from the Indian Ocean into the western Pacific, with slower speed and stronger amplitude compared to westerly QBO (WQBO) periods. This change to MJO activity during different QBO phases is strong and statistically significant, especially in DJF: The influence of the QBO is larger than that of El Niño Southern Oscillation phases over the tropical Indo‐Pacific and seems to be a dominant driver of interannual MJO variability (e.g., Son et al., 2017). In seasons outside of boreal winter, no strong QBO‐MJO relationship has been observed.

One plausible explanation of the robust QBO‐MJO connection is QBO‐induced changes to static stability. Both observational studies (Hendon & Abhik, 2018; Nishimoto & Yoden, 2017; Son et al., 2017; Yoo & Son, 2016; Zhang & Zhang, 2018) and idealized cloud‐resolving model studies (Martin et al., 2019; Nie & Sobel, 2015) have argued that QBO‐related temperature anomalies near the tropical tropopause layer (induced via thermal wind constraints) are the key driver of MJO activity change: During EQBO, negative temperature anomalies reduce the static stability between the upper troposphere and lower stratosphere in the tropical Indo‐Pacific, which acts to promote stronger deep convection associated with the MJO. Using reconstructions of QBO and MJO indices back to 1905, Klotzbach et al. (2019) showed that the observed QBO‐MJO relationship has only emerged since the 1980s. They suggest that this emergence may be driven by the recent warming trend in the upper troposphere and cooling trend in the lower stratosphere, which together act to further reduce the stability in the equatorial tropopause, thus making the MJO more active in EQBO winter. While this hypothesis has become increasingly well supported, the precise details of this mechanism are still not clear or settled.

The QBO‐MJO relationship has been further explored in studies of how the QBO impacts MJO predictability, where studies generally have reached a consensus: Namely, the boreal winter MJO is more predictable in EQBO than in WQBO (Abhik & Hendon, 2019; Lim et al., 2019; Marshall et al., 2017; Wang et al., 2019). By comparing multimodel reforecasts from the WMO Subseasonal to Seasonal Prediction project (S2S, Vitart et al., 2017), studies have shown that higher MJO prediction skill during EQBO is not simply due to more initially strong MJO events; rather, the increase in skill seems to stem from a better organized MJO during the forecast (Abhik & Hendon, 2019; Lim et al., 2019; Marshall et al., 2017). However, also using the multimodel S2S reforecasts, Wang et al. (2019) argued that while the QBO‐MJO skill relationship strongly depends on the observed QBO and its associated MJO initial condition, it is weakly dependent on the forecasted QBO. This indicates that the direct influence of the forecasted QBO in the model may not be the main factor contributing to the QBO‐MJO skill relationship.

The objective of this study is to examine whether the QBO‐MJO skill relationship found in the previous studies is robust in the reforecasts from the newly launched Subseasonal Experiment (SubX)—a research‐to‐operations project (Kirtman et al., 2017; Pegion et al., 2019) – and from NCAR Community Earth System Model v1 (CESM1) reforecasts. SubX consists of multiple models from the current generation prediction systems (Table 1). NCAR‐CESM1 reforecasts were carried out using the SubX protocol, but NCAR‐CESM1 does not produce real‐time forecasts. The MJO prediction skills in SubX reforecasts are comparable to those in the S2S project (Kim et al., 2019).

**Table 1:** Nine Subseasonal Reforecast Models Considered in This Study

Models	Vertical levels (upper boundary; hPa)	MJO events		Initialization interval (ensemble members)	Years (reforecast period)
		EQBO (6 years)	WQBO (7 years)		
NCAR‐CESM1	L30 (2.0) L46 (0.3)	58	48	1/week (10)	17 years (1999–2015)
NCEP‐GEFS^*^	L64 (0.2)			1/week (11)	
ESRL‐FIM^*^	L64 (0.5)			1/week (4)	
RSMAS‐CCSM4^*^	L26 (3.0)			1/week (3)	
Navy‐ESPC^*^	L50 (0.04)	234	210	4/week (1)	
NASA‐GEOS5^*^	L72 (0.01)	83	84	Every 5 days (4)	
ECMWF‐Cy43r3^**^	L91 (0.01)	116	102	2/week (11)	
KMA/UKMO‐GloSea5^**^	L85 (0.005)	50	49	4/month (3)	18 years (1993–2010)

*Note*. One star (^*^) denotes SubX models; Two stars (^**^) denote S2S models. NCAR‐CESM1 = National Center for Atmospheric Research Community Earth System Model version 1; NCEP‐GEFS = National Centers for Environmental Prediction Environmental Modeling Center Global Ensemble Forecast System; ESRL‐FIM = NOAA Earth System Research Laboratory Flow‐Following Icosahedral Model; RSMAS‐CCSM4 = Community Climate System Model version 4 run at the University of Miami Rosenstiel School for Marine and Atmospheric Science; Navy‐ESPC = Naval Research Laboratory Navy Earth System Prediction Capability; NASA‐GEOS5 = National Aeronautics and Space Administration Global Modeling and Assimilation Office Goddard Earth Observing System‐5; ECMWF‐Cy43r3 = European Centre for Medium‐Range Weather Forecasts version Cy43r3; KMA/UKMO‐GloSea5 = Korea Meteorological Administration‐UK Met Office coupled Global Seasonal forecast.

In addition to considering a new database of models than other studies, an advantage of using the SubX and NCAR‐CESM1 reforecasts is that the QBO‐MJO skill relationship could be sensitive to the period considered (i.e., Klotzbach et al., 2019) or oscillate on longer timescales (e.g., Wang et al., 2019; who found decadal dependence of the QBO impact on boreal summer intra‐seasonal convection). Because the S2S total reforecast periods range from 11 to 30 years depending on the forecast system, the selected number of WQBO years, for example, ranges from 5 to 15 years (Lim et al., 2019). Wang et al. (2019) used the common period among S2S models, which encompasses 11 years of the total reforecast period (1999–2010). In this regard, one of the advantages of using SubX and NCAR‐CESM1 models is that the reforecasts are performed for 17 years over a common period from 1999 to 2015, making the results less sensitive to the QBO year selection and spanning a longer period than some other studies.

In addition to SubX and NCAR‐CESM1 models, this study also includes two S2S models (ECMWF‐Cy43r3 and KMA/UKMO‐GloSea5). These two S2S models are selected because (i) they have relatively high MJO prediction skill among the S2S models (Lim et al., 2019; Vitart, 2017), (ii) they consist of a relatively long reforecast period (≥17 years), (iii) they have the highest vertical resolution with highest top among S2S models (Lim et al., 2019), and (iv) the comparison with the SubX models will complement the findings by Lim et al. (2019) and Wang et al. (2019) who used S2S models. Finally, in addition to the multimodel comparison, a comparison is made between the high‐top (46‐level, hereafter L46) and low‐top (30‐level, hereafter L30) version of the NCAR‐CESM1, in order to support our general conclusions and further understand the impact of the forecasted QBO on MJO prediction. The reforecasts and validation data sets are described in section 2. QBO‐MJO skill relationship in subseasonal models is assessed in section 3. NCAR‐CESM1 reforecasts experiments are compared in section 4, followed by summary and discussion in section 5.

## 2 Data and Method

The ECMWF Interim Reanalysis product (ERAI, Dee et al., 2011) and the NOAA Advanced Very High‐Resolution Radiometer Outgoing Longwave Radiation (OLR) (Liebmann & Smith, 1996) are used to examine the observed MJO and QBO. These data sets are used to calculate the Real‐time Multivariate MJO (RMM, Wheeler & Hendon, 2004) index using the 200 and 850 hPa daily zonal wind from ERAI and daily OLR data from NOAA; these are referred to as “observation” for brevity. The QBO is defined using the ERAI monthly‐mean zonal‐mean zonal wind at 50 hPa (U50) averaged over 10°S–10°N. WQBO and EQBO are defined when the DJF averaged U50 anomaly is, respectively, larger than or less than 0.5 standard deviation during 1979 to 2015, a methodology following recent studies (Abhik & Hendon, 2019; Lim et al., 2019; Marshall et al., 2017; Yoo & Son, 2016). Over the reforecast period, the selected EQBO winters are 1996/1997, 1998/1999, 2001/2002, 2003/2004, 2005/2006, 2007/2008, 2012/2013, and 2014/2015 (where, for example, 2014/2015 refers to the mean of December 2014 through February 2015). Selected WQBO winters are 1995/1996, 1997/1998, 1999/2000, 2002/2003, 2004/2005, 2006/2007, 2008/2009, 2010/2011, and 2013/2014. Selected QBO winters are consistent with previous studies (e.g., Hendon & Abhik, 2018; Lim et al., 2019). Note that the KMA/UKMO‐GloSea5 uses some QBO years differently from other models due to the different reforecast period (Table 1). Here, the number of QBO years (six EQBO and seven WQBO) are the consistent in each reforecast. We evaluate the QBO‐MJO prediction skill relationship during DJF (reforecasts initialized between 1 December and 28 February).

Table 1 provides information about the nine subseasonal reforecast models considered in this study (five SubX, two S2S, and two versions of NCAR‐CESM1 with differing model levels in the stratosphere), including the number of selected QBO and MJO events, initialization interval, ensemble size, vertical resolution, and reforecast period. References in Vitart et al. (2017) and Pegion et al. (2019) provide more detailed information of each model's configurations. All models are initialized at least once a week and have reforecasts out to a minimum of 32 forecast lead days. Forecast and observed anomalies are, respectively, computed with regards to model or observed climatologies as a function of forecast lead days (detailed processes in Pegion et al., 2019). All reforecasts and observations are interpolated to a 1° longitude and 1° latitude grid.

In both observation and reforecasts, “MJO events” are identified when the normalized value of the observed RMM amplitude (defined as $\sqrt{\mathrm{RMM}1^{2}+\mathrm{RMM}2^{2}}$) exceeds 1.5 at initial Day 0; about 65% of total days are classified as MJO events in observation. Numbers of selected MJO events by QBO phases in each model are listed in Table 1. Note that the selected MJO events differ among models due to different reforecast period and initialization frequency. Finally, because of the similar MJO characteristics and prediction skill in NCAR‐CESM1 L30 and L46 (section 4), we combine them into a single 20‐member ensemble by taking the 10 members from each for the QBO‐MJO skill relationship in section 3. The difference between the L30 and L46 versions is discussed in more detail in section 4. All results shown in this study for all models are based on ensemble mean.

## 3 QBO‐MJO Skill Relationship in the Subseasonal Reforecasts

**Figure 1:**
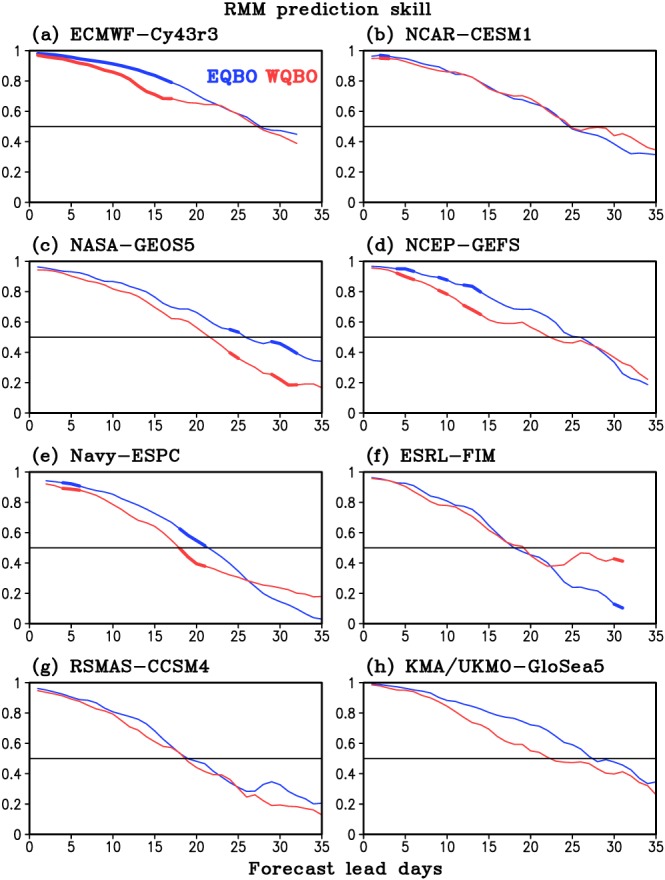
RMM skill (bivariate correlation coefficient) of MJO events during EQBO (blue) and WQBO (red) boreal winters (DJF) as a function of forecast lead days for eight different prediction systems. Horizontal line denotes correlation coefficient of 0.5. Thick lines indicate days when the RMM skill difference between EQBO and WQBO is statistically significant at 95% confidence level based on 100,000 sample bootstrap.

To evaluate the MJO prediction skill, the RMM bivariate correlation coefficients (RMM skill, hereafter) are computed between the predicted and observed RMM1 and RMM2 indices as a function of forecast lead days for the selected MJO events. The bivariate correlation coefficient (BCOR) is calculated as:

$$\text{BCOR}\left(\tau \right)=\frac{\sum_{t=1}^{t=N}\left[a_{1}\left(t\right)b_{1}\left(t,\tau \right)+a_{2}\left(t\right)b_{2}\left(t,\tau \right)\right]}{\sqrt{\sum_{t=1}^{t=N}\left[a_{1}^{2}\left(t\right)+a_{2}^{2}\left(t\right)\right]}\sqrt{\sum_{t=1}^{t=N}\left[b_{1}^{2}\left(t,\tau \right)+b_{2}^{2}\left(t,\tau \right)\right]}}$$

where *a*_1_(*t*) and *a*_2_(*t*) are the observed RMM1 and RMM2 at time *t*, and *b*_1_(*t*, *τ*) and *b*_2_(*t*, *τ*) are the respective forecasts of RMM1 and RMM2 for time *t* with a lead time of *τ* days. *N* is the number of MJO events (Table 1). Figure 1 shows the RMM skill as a function of forecast lead days during EQBO and WQBO winter for each model. Thick lines indicate when RMM skill difference between EQBO and WQBO is statistically significant at 95% confidence level determined by a bootstrap method as follows: First, the RMM skill is constructed by selecting random years during the reforecast period with same number of each QBO years. For example, for ECMWF‐Cy43r3, six (group A) and seven (group B) boreal winters were randomly sampled between 1999 and 2015 without overlapping of years. Then, the RMM skill is computed between sampled reforecasts and corresponding observation in Groups A and B, respectively, as a function of forecast lead days (as in Figure 1). The RMM skill difference between Groups A and B is then computed. This process is repeated 100,000 times with replacement to obtain a probability distribution function of RMM skill difference. The confidence intervals are determined by ranking the results of the 100,000 bootstrapping tests and finding the 2.5th and 97.5th percentiles of the distribution. The RMM skill difference between EQBO and WQBO is significant at 95% confidence level if the skill difference lies outside the 2.5th to 97.5th percentile.

Out of eight models, five (ECMWF‐Cy43r3, NASA‐GEOS5, NCEP‐GEFS, Navy‐ESPC, and KMA/UKMO‐GloSea5) show higher RMM skill during EQBO than WQBO up to 25 days, consistent with previous studies. However, none of the models show significant RMM skill difference constantly during the entire forecast. Only the ECMWF‐Cy43r3 shows an extended, unbroken period of significant RMM skill difference, and even there it is only out to 17 days. Note that Weeks 3–4 (15–28 days) is the main target of subseasonal forecast as it is beyond weather time scales (i.e., beyond around 7–10 days), and at this stage, no model has a significant RMM skill difference. NASA‐GEOS5, Navy‐ESPC, and ESRL‐FIM have only a few forecast lead days when the skill difference becomes significant. Lowering the confidence level to 90% only adds 1–3 days of significant skill difference in some models (not shown).

**Figure 2:**
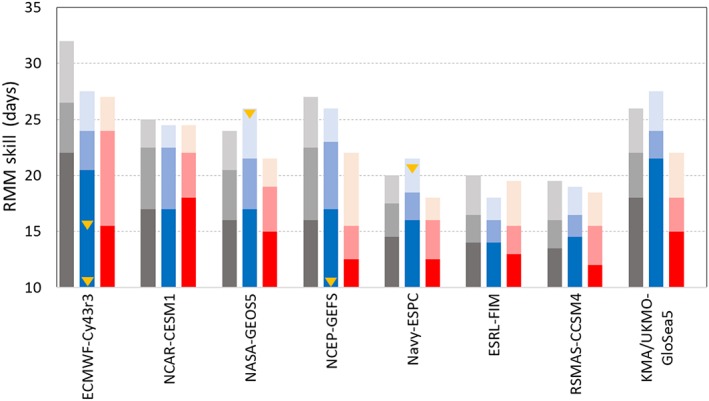
Forecast lead days when RMM skill reaches 0.7 (dark), 0.6 (medium), and 0.5 (light) in all (gray), EQBO (blue), and WQBO (red) winters. Yellow triangles indicate when RMM skill difference between EQBO and WQBO is statistically significant at 95% confidence level at the forecast lead Days 10, 15, 20, and 25 based on Figure 1.

Figure 2 summarizes Figure 1 by showing the forecast lead days when RMM skill of selected MJO events for each model reaches 0.7, 0.6, and 0.5, during EQBO, WQBO, and all winters. Consistent with previous studies (Lim et al., 2019; Wang et al., 2019), ECMWF‐Cy43r3 shows the highest skill among models for all winters, in which the forecast days of RMM skill 0.5 exceeds 30 days. It is obvious that RMM skill is higher in EQBO winter than WQBO in most models. However, only two models (NASA‐GEOS5 and Navy‐ESPC) show statistically significant RMM skill difference beyond 20 days. Lim et al. (2019, their Figure 3) also showed that out of 10 S2S models, only one model (China Meteorological Administration) has significant skill difference (at 95% confidence level) beyond 20 days, although that model has low MJO prediction skill.

**Figure 3:**
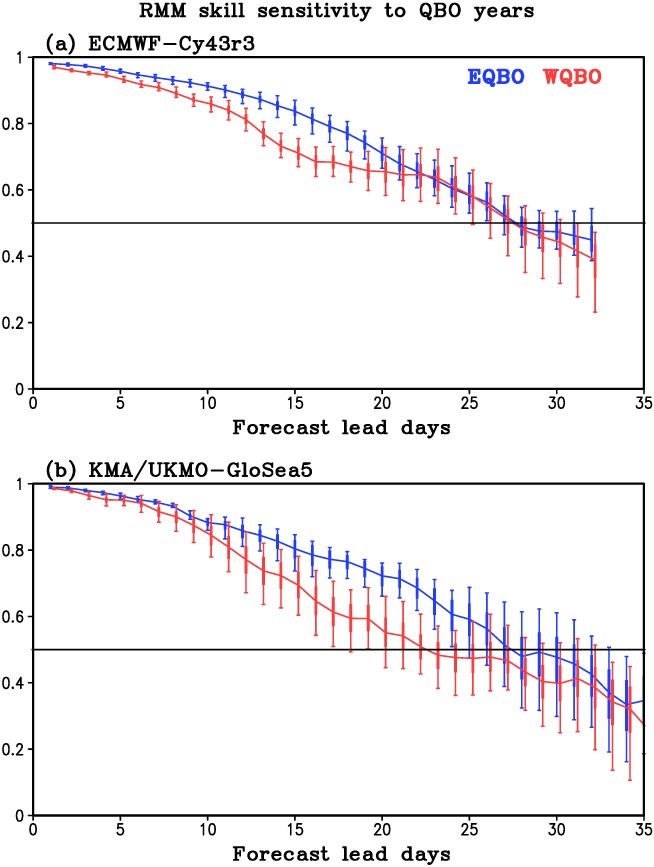
The box‐whisker plots represent the spread of RMM skill between 15 EQBO (blue) and 21 WQBO (red) combinations for (a) ECMWF‐Cy43r3 and (b) KMA/UKMO‐GloSea5. The box outlines the ±1.0 standard deviation of RMM skill, and whiskers indicate the minimum and maximum values. The contour lines are the RMM skills shown in Figure 1.

As mentioned, the QBO‐MJO skill relationship could be sensitive to the period considered. The selected WQBO years, for example, range from 5 to 15 years in S2S models depending on the forecast system (Lim et al., 2019). To account for this possibility, we test the sensitivity of RMM skill to the number of selected QBO years. All possible combinations are obtained by taking all subsets of 4 out of 6 EQBO years and 5 out of 7 WQBO years: 15 subsets of EQBO and 21 subsets of WQBO. These years (four EQBO and five WQBO) approximately match the number of QBO years in the S2S models that have the shortest reforecast period. RMM skill is then calculated for each subset (15 EQBO and 21 WQBO) in both ECMWF‐Cy43r3 and KMA/UKMO‐GloSea5, which have relatively high RMM skill (Figure 2) and the highest vertical resolution with the highest upper boundary among the reforecasts (Table 1). The conclusions of the following analysis hold for if all SubX models are used (not shown). Figure 3 shows the variability of RMM skill in these subsets. A large spread of RMM skill is evident for both models. Forecast lead days where the whiskers do not overlap are indicative of days where EQBO and WQBO have significant RMM skill difference. In both models, large spread is shown in Weeks 3–4 lead time where the RMM skill in WQBO overlaps or covers the skill range in EQBO after Week 3 (Figures 3a and 3b). In KMA/UKMO‐GloSea5, the spread become large within 10 days, especially in WQBO phase (Figure 3b). This result indicates that the QBO‐MJO skill relationship is sensitive to QBO year selection, which supports why most of models show insignificant QBO‐MJO skill relationship—a result that will be examined further in the next section.

## 4 NCAR‐CESM1 Reforecast Experiments: High‐Top Versus Low‐Top Model

The QBO‐MJO skill relationship observed in forecast models can be influenced by the observed and predicted MJO characteristics as well as the QBO‐MJO interaction during the forecast. Prediction skill, in general, can further be influenced by model configuration and forecast experimental design choices, such as ensemble size, initialization frequency, and forcast period. Therefore, comparison of QBO‐MJO skill relationship in different forecast systems further complicates the interpretation of QBO‐MJO skill results.

To better understand the direct influence of the forecasted QBO on MJO prediction, or lack thereof, a set of reforecast experiments is conducted with NCAR‐CESM1 in which everything is consistent except the vertical levels, vertical resolution—which includes a lower‐top (L30) and a higher‐top (L46) configuration of the Community Atmosphere Model v5 (CAM5)—and the gravity wave parametrization. L30 has 30 vertical levels and a model top at ~2 hPa (Neale et al., 2012) with 8 vertical levels from 100 to 2 hPa. L46 has 46 vertical levels and a model top at 0.3 hPa (Richter et al., 2014a) with 23 vertical levels from 100 to 0.3 hPa. The L46 includes nonorographic gravity wave parameterization which leads to a more realistic QBO simulation (Richter et al., 2014a). A spectral element dynamical core (Dennis et al., 2012) with a horizontal resolution of approximately 100 km is used for both L46 and L30. Both L46 and L30 reforecasts each have a 10‐member ensemble, initialized once a week at the same date with same initial conditions, and integrated over the common period from 1999 to 2015, following the SubX protocol (Pegion et al., 2019). Therefore, if there are significant changes in MJO detected during the forecast, those changes must stem from the effect of vertical resolution, gravity wave parametrization, and/or an altered QBO simulation. ERAI is compared with the predictions over the same period (1999–2015). Output from the L30 and L46 are interpolated onto 17 vertical pressure levels to match them with the ERAI levels.

**Figure 4:**
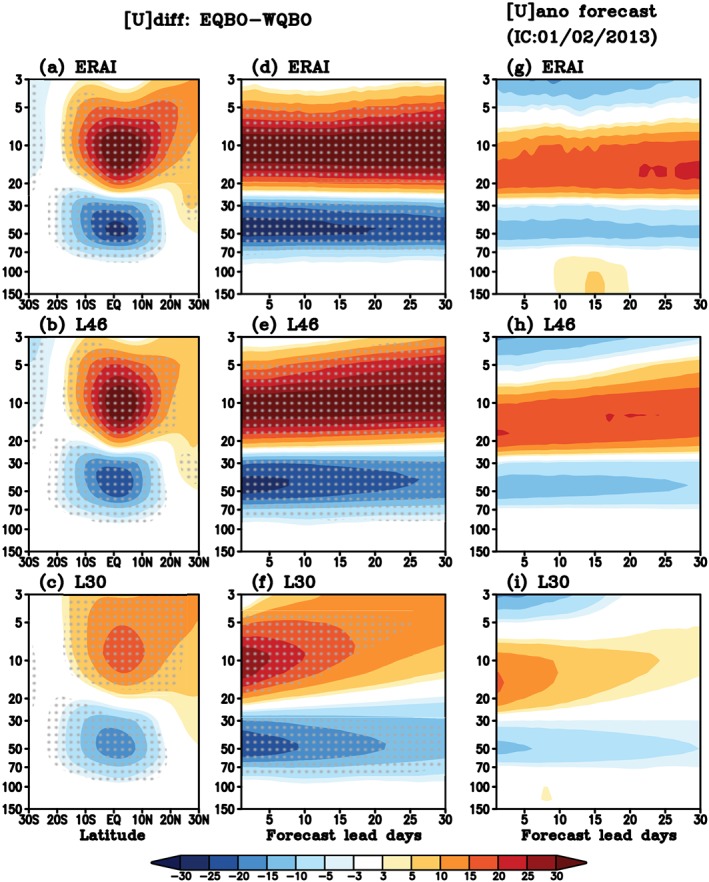
(Left) Composite of zonal‐mean zonal wind (m/s) difference between EQBO and WQBO winters ([*U*]_*diff*_) for (a) January in ERAI, and a 30 day average for (b) L46 and (c) L30 reforecasts initialized in early January, and (middle) [*U*]_*diff*_ averaged over 5°S–5°N as a function of forecast lead days. Stipples mark significant difference at the 95% confidence level. (Right) Zonal‐mean zonal wind anomaly (m/s) averaged over 5°S–5°N ([*U*]_*ano*_) as a function of forecast lead days starting from 2 January 2013.

Compared to L30, L46 better simulates an internally generated QBO with more realistic structure and statistics, as well as stratospheric sudden warmings (Richter et al., 2014b). To examine the QBO simulations in both model versions, zonal‐mean zonal wind composite of EQBO minus WQBO ([*U*]_*diff*_, hereafter) is displayed in Figure 4, along with the January mean [*U*]_*diff*_ in ERAI. Predicted [*U*]_*diff*_ is calculated with the reforecasts initialized at early January (between 1 and 7 of each January, depending on the year) and averaged over 1 to 30 forecast lead days, which roughly mimics the observed January mean. Dotted areas indicate statistically significant difference between EQBO and WQBO exceeding the 95% confidence level according to the two‐tailed Student's *t* test. Here, we chose January only to be consistent with the following discussion on QBO‐MJO skill; including additional months (e.g., December or February) does not alter the [*U*]_*diff*_ patterns shown in Figure 4a significantly (not shown).

**Figure 5:**
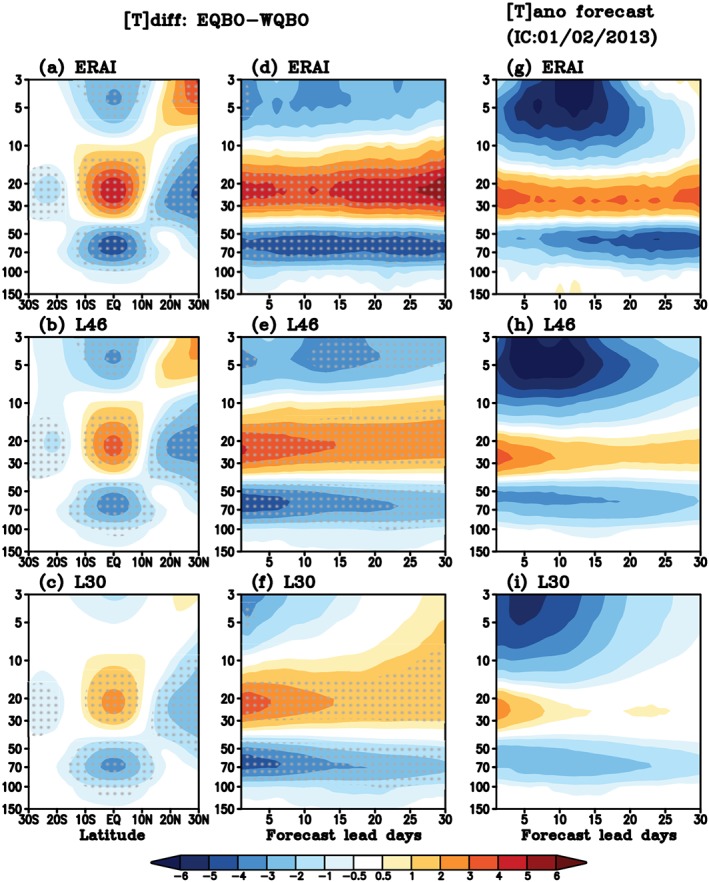
Same as Figure 4, except for temperature (K).

The ERAI (Figure 4a) shows a significant EQBO‐WQBO signal with the prevailing easterlies between 100 to 30 hPa and westerlies above, associated with consistent temperature differences ([*T*]_*diff*_, Figure 5a) in keeping with thermal wind balance. Changes in [*U*]_*diff*_ are also compared, as a function of lead days, in ERAI and reforecasts (Figures 4d–4f). Note that the ERAI starts at 1 January and does not exactly correspond to the dates of the reforecast. Overall, L46 (Figures 4b and 4e) predicts a reasonable [*U*]_*diff*_ pattern, although the magnitude of wind is slightly weaker than the observed. L30 (Figures 4c and 4f) also captures a [*U*]_*diff*_ pattern but with substantially weaker magnitude and strong biases especially in the upper stratosphere (above 10 hPa). This deficiency in L30 reflects the importance of high stratospheric resolution, and gravity wave drag needed to maintain the QBO signal. [*U*]_*diff*_ shown in Figures 4a–4f range up to ~±30 m s^−1^ in ERAI and L46 but only up to ~±20 m s^−1^ in L30. The patterns and relative magnitudes for [*T*]_*diff*_ are consistent with [*U*]_*diff*_, with weaker magnitudes in L30 than L46 (Figures 5a–5f).

**Figure 6:**
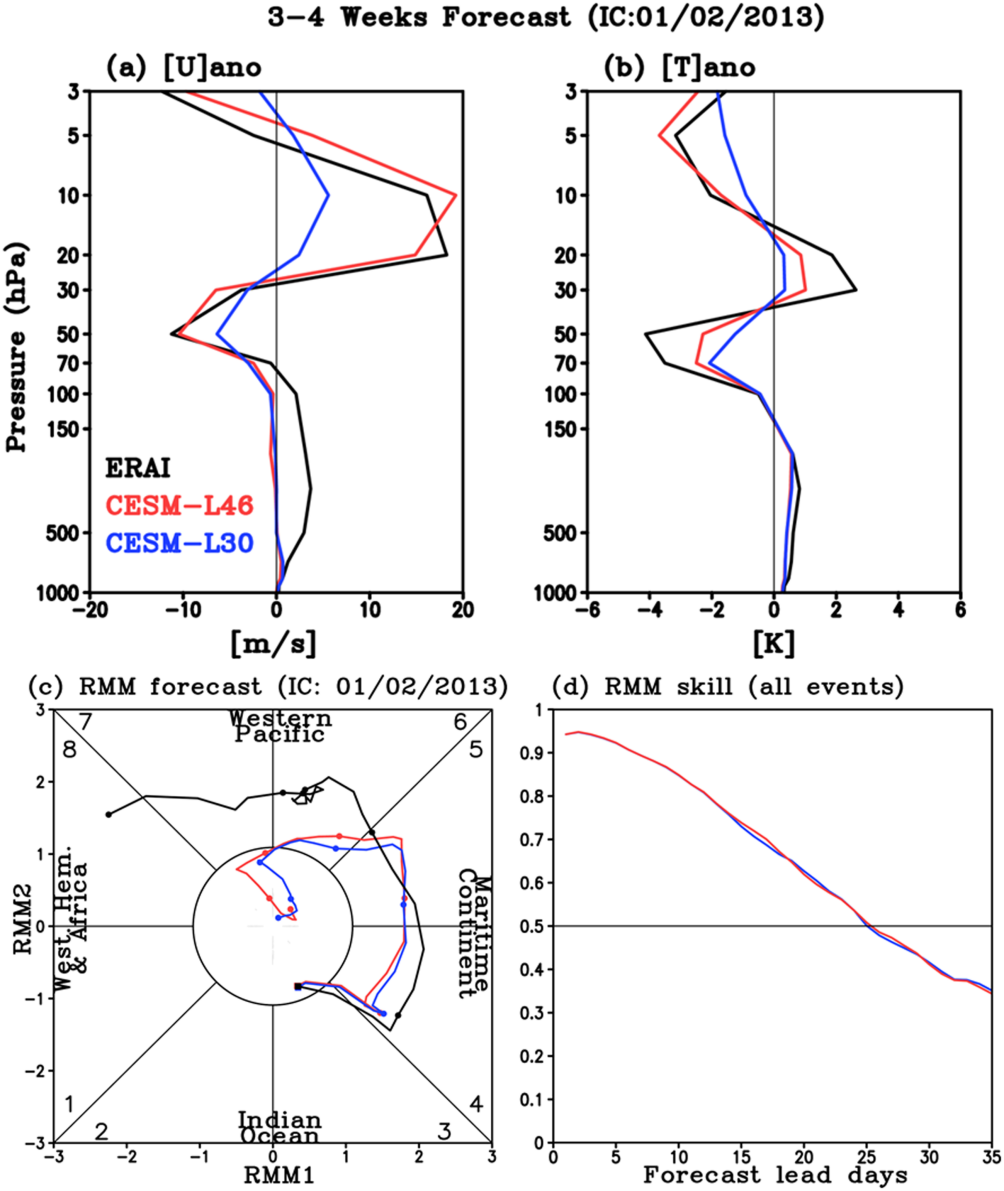
3–4 weeks averaged (a) [*U*]_*ano*_, (b) [*T*]_*ano*_, and (c) RMM forecasts starting from 2 January 2013 for observation (black), L46 (red), and L30 (blue). Dots in (c) represent every 5 days from the forecast starting date (square). (d) RMM skill for all boreal winter MJO events over the 1999–2015 hindcast period.

To investigate the QBO's impact on MJO prediction, we focus on January 2013 when robust QBO (Figure 4g) and MJO (Figure 6c) were observed simultaneously. Figure 4g shows the observed zonal wind anomaly relative to January climatology averaged zonally and between 5°S–5°N ([*U*]_*ano*_, hereafter) starting from 2–31 January 2013 (30 days). A clear sustained EQBO signal is observed, with the maximum easterly anomalies centered near 50 hPa and overlaying westerly anomalies between 10 and 30 hPa associated with the next descending WQBO phase for the end of 2012/2013 winter. The apparent EQBO signal can be seen in the vertical profile of [*U*]_*ano*_ for Weeks 3–4 average (15 to 28 days average) from the start of January 2013 (Figure 6a). Here again, the Weeks 3–4 average is chosen to examine the prediction skill of models at subseasonal time scales.

Previous studies have argued that the QBO related temperature change near the tropical tropopause layer is the key driver of MJO activity change (Hendon & Abhik, 2018; Klotzbach et al., 2019; Martin et al., 2019). To examine the temperature changes for January 2013, evolution of the [*T*]_*ano*_ and Weeks 3–4 averaged vertical profile are compared (Figures 5g and 6b). The observed [*T*]_*ano*_ shows a typical EQBO profile with negative temperature anomalies within the upper troposphere/lower stratosphere (30–100 hPa) consistent with the wind profile. Such anomalous temperature structure reduces static stability in the upper troposphere/lower stratosphere, thus making the environment more favorable for tropical deep convection. When the EQBO‐induced negative temperature anomaly extends from the stratosphere into the upper troposphere, it constructively adds to the destabilization produced by the MJO deep convection, making the MJO stronger (e.g., Hendon & Abhik, 2018; Son et al., 2017; Zhang & Zhang, 2018). The observed MJO (Figure 6c, black line) shows a clear eastward propagation starting from the Indian Ocean on 2 January 2013, through the Maritime Continent and into the western Pacific during the month. Note that the observed RMM amplitude is strong when it propagates from the Maritime Continent into the Pacific.

In the reforecasts, L46 keeps the initial EQBO signal in [*U*]_*ano*_ throughout the entire month with a comparable magnitude of the observed, especially at Weeks 3–4 (Figures 4h and 6a). The [*U*]_*ano*_ in L30 (Figure 4i) shows similar pattern to L46 at the beginning of the reforecast, because they are initialized with the same data. However, the initial signal weakens rapidly within 2 weeks over the entire stratosphere (Figures 4i and 6a), especially above 100 hPa where the long‐term mean bias develops quickly in both zonal wind and temperature (not shown). Compared to the observed anomalies, both models simulate weaker [*T*]_*ano*_ in general (Figures 5 and 6b) and in particular within the lower stratosphere (between 50 to 100 hPa): The very region that is likely most important for the QBO‐MJO interaction (e.g., Son et al., 2017). In L30, temperature anomalies become even weaker than L46. At 50 hPa, the observed Weeks 3–4 [*T*]_*ano*_ is approximately −4 K. In both model configurations, the magnitude of the [*T*]_*ano*_ minimum is weaker: For L46, it is reduced by less than 50% whereas for L30, it is reduced by ~80% (Figure 6b), reflecting the rapid decay of the QBO temperature signal after a week (Figure 5i).

Since the QBO signal decays faster in the L30 than L46, one may expect to see differences in MJO prediction if the stratosphere influences the tropospheric convection during the forecast. However, predicted RMM indices are almost identical between the two models for January 2013 event (Figure 6c). Both models predict very similar MJO propagation to each other, while also having weaker amplitude than the observed. Figure 6c is a prediction of only one MJO event, but the RMM skill for MJO events over the total 17 years (1999–2015) of DJF is almost identical between the two models (Figure 6d). Furthermore, the biases in amplitude and phase of MJO are almost identical between two models (not shown). Because of such similar MJO characteristics, we combined each of 10 ensembles from L30 and L46 to obtain the maximum MJO prediction skill in section 3 and in Kim et al. (2019). This lack of a difference in skill further implies that the better simulated QBO in L46 does not translate into better MJO prediction for NCAR‐CESM1, challenging the notion that the stratosphere has an impact on MJO prediction after the simulation is initialized.

## 5 Summary and Discussion

The impact of the QBO on MJO prediction is evaluated in the SubX, S2S, and NCAR‐CESM1 subseasonal reforecasts experiments. When MJO prediction skill is compared during DJF, RMM prediction skill for initially strong MJO events is generally higher in EQBO than WQBO, consistent with previous studies. Importantly though, for nearly all models, the RMM skill difference is not statistically significant. The insignificant QBO‐MJO skill relationship is further confirmed by comparing two reforecast experiments with high‐top (L46) versus low‐top (L30) versions of CAM5 of NCAR‐CESM1. Although the L46 model predicts a more realistic QBO than the L30 in both wind and temperature fields, the predicted MJO events in both versions of the model are almost identical. This indicates that the QBO may not directly influence the MJO during the simulation, consistent with Wang et al. (2019), or that the QBO and/or MJO are not sufficiently well represented even in the L46 model to produce the correct QBO‐MJO interactions.

The insignificant QBO‐MJO skill relationship shown in the SubX, S2S, and NCAR‐CESM1 reforecast experiment could be due to several factors. First, the model's QBO signal in the lower stratosphere/upper troposphere may be too weak for the MJO to realize the QBO forcing during the forecasts. For example, although the vertical temperature profile in L46 is closer to the observed (Figures 5 and 6b), the tropopause static stability (−𝑑𝑇/𝑑𝑝) is still smaller than the observed, indicating that the QBO is not sufficiently well predicted in the L46. Additionally, though the QBO is better simulated at upper levels in L46, signals around the tropopause (~100 hPa) are comparable between the two versions. Martin et al. (2019) found in an idealized cloud‐resolving model that the MJO response to the QBO was quite sensitive to the magnitude and height of the QBO anomaly, such that small differences around the tropopause may have large effects on the QBO‐MJO link.

Second, the MJO loses its amplitude very quickly in the models considered here, a common problem in forecast models (Kim et al., 2018; Kim et al., 2019; Vitart, 2017). It may be the case that the MJO signal is too weak to be modulated by the QBO. If the models simulate both a weaker QBO and a weaker MJO together, both biases may weaken the destabilization effect, thus limit further QBO‐MJO interaction during the forecast. Third, in addition to the systematic biases in simulated QBO and MJO, models may be missing key processes driving observed QBO‐MJO connection (Lee & Klingaman, 2018); in this regard, more observational studies pinpointing particular mechanisms would be of use. Relatedly, the vertical resolution in current models, especially near the tropopause, may not be sufficient to resolve the key processes. This can be further tested by using higher‐top model or finer vertical resolution (Garcia & Richter, 2019).

This study is not opposed to the recent evidence on the observed QBO‐MJO relationship argument; our focus here is primarily on whether initialized forecast models show significant differences in MJO prediction under various QBO phases and further whether the predicted QBO in models affects the MJO during the forecast. When the stratospheric impact on MJO prediction is evaluated, caution needs to be taken because the smaller sample size of QBO and MJO events than in observation makes the result sensitive to the choice of period and events. Further, current models have strong biases in predicting local MJO convection generally, especially when MJO starts from the Indian Ocean and propagates through the Maritime Continent into the western Pacific (e.g., Kim et al., 2018; 2019). Because the MJO convection itself is often not well predicted in the models, it is hard to expect a realistic stratospheric modulation on the MJO, because MJO is primarily a tropospheric phenomenon.

## References

[jgrd55919-bib-0001] Abhik, S. , & Hendon, H. H. (2019). Influence of the QBO on the MJO during coupled model multiweek forecasts. Geophysical Research Letters, 46(15), 9213–9221. 10.1029/2019GL083152

[jgrd55919-bib-0002] Baldwin, M. P. , Gray, L. J. , Dunkerton, T. J. , Hamilton, K. , Haynes, P. H. , Randel, W. J. , Holton, J. R. , Alexander, M. J. , Hirota, I. , Horinouchi, T. , Jones, D. B. A. , Kinnersley, J. S. , Marquardt, C. , Sato, K. , & Takahashi, M. (2001). The quasi‐biennial oscillation. Reviews of Geophysics, 39(2), 179–229. 10.1029/1999RG000073

[jgrd55919-bib-0003] Dee, D. P. , Uppala, S. M. , Simmons, A. J. , Berrisford, P. , Poli, P. , Kobayashi, S. , Andrae, U. , Balmaseda, M. A. , Balsamo, G. , Bauer, P. , Bechtold, P. , Beljaars, A. C. M. , Van de Berg, L. , Bidlot, J. , Bormann, N. , Delsol, C. , Dragani, R. , Fuentes, M. , Geer, A. J. , Haimberger, L. , Healy, S. B. , Hersbach, H. , Hólm, E. V. , Isaksen, L. , Kållberg, P. , Köhler, M. , Matricardi, M. , McNally, A. P. , Monge‐Sanz, B. M. , Morcrette, J. J. , Park, B. K. , Peubey, C. , De Rosnay, P. , Tavolato, C. , Thépaut, J. N. , & Vitart, F. (2011). The ERA‐Interim reanalysis: Configuration and performance of the data assimilation system. Quarterly Journal of the Royal Meteorological Society, 137(656), 553–597. 10.1002/qj.828

[jgrd55919-bib-0004] Dennis, J. M. , Edwards, J. , Evans, K. J. , Guba, O. , Lauritzen, P. H. , Mirin, A. A. , St‐Cyr, A. , Taylor, M. A. , & Worley, P. H. (2012). CAM‐SE: A scalable spectral element dynamical core for the Community Atmosphere Model. The International Journal of High Performance Computing Applications, 26(1), 74–89. 10.1177/1094342011428142

[jgrd55919-bib-0005] Garcia, R. R. , & Richter, J. H. (2019). On the momentum budget of the quasi‐biennial oscillation in the whole atmosphere community climate model. Journal of the Atmospheric Sciences, 76(1), 69–87. 10.1175/JAS-D-18-0088.1

[jgrd55919-bib-0006] Hendon, H. H. , & Abhik, S. (2018). Differences in vertical structure of the Madden‐Julian Oscillation associated with the quasi‐biennial oscillation. Geophysical Research Letters, 45(9), 4419–4428. 10.1029/2018GL077207

[jgrd55919-bib-0007] Kim, H. , Janiga, M. A. , & Pegion, K. (2019). MJO propagation processes and mean biases in the SubX and S2S reforecasts. Journal of Geophysical Research: Atmospheres, 124(16), 9314–9331. 10.1029/2019JD031139 PMC6988457 32025452

[jgrd55919-bib-0008] Kim, H. , Vitart, F. , & Waliser, D. E. (2018). Prediction of the Madden–Julian Oscillation: A Review. Journal of Climate, 31(23), 9425–9443. 10.1175/JCLI-D-18-0210.1

[jgrd55919-bib-0009] Kirtman, B. P. , Pegion, K. , DelSole, T. , Tippett, M. , Robertson, A. W. , Bell, M. , Burgman, R. , Lin, H. , Gottschalck, J. , Collins, D. C. , Li, W. , Sinsky, E. , Guan, H. , Zhu, Y. , Becker, E. J. , Lajoie, E. , MacRitchie, K. , Min, D. , Fu, R. , Achuthavarier, D. , Koster, R. , Marshak, L. , Lin, H. , Denis, B. , Barton, N. , Green, B. W. (2017). The Subseasonal Experiment (SubX). IRI Data Library. 10.7916/D8PG249H

[jgrd55919-bib-0010] Klotzbach, P. , Abhik, S. , Hendon, H. , Bell, M. , Lucas, C. , Marshall, A. , & Oliver, E. (2019). On the emerging relationship between the stratospheric quasi‐biennial oscillation and the madden‐julian oscillation. Scientific Reports, 9(1), 2981 10.1038/s41598-019-40034-6 30814656 PMC6393487

[jgrd55919-bib-0011] Lee, J. C. , & Klingaman, N. P. (2018). The effect of the quasi‐biennial oscillation on the Madden‐Julian oscillation in the Met Office unified model global ocean mixed layer configuration. Atmospheric Science Letters, 19(5), e816 10.1002/asl.816

[jgrd55919-bib-0012] Liebmann, B. , & Smith, C. A. (1996). Description of a complete (interpolated) outgoing longwave radiation dataset. Bulletin of the American Meteorological Society, 77, 1275–1277.

[jgrd55919-bib-0014] Lim, Y. , Son, S.‐W. , Marshall, A. G. , Hendon, H. H. , & Seo, K.‐H. (2019). Influence of the QBO on MJO prediction skill in the subseasonal‐to‐seasonal prediction models. Climate Dynamics, 1–15.

[jgrd55919-bib-0015] Madden, R. A. , & Julian, P. R. (1971). Detection of a 40–50 day oscillation in the zonal wind in the tropical Pacific. Journal of the Atmospheric Sciences, 28(5), 702–708. 10.1175/1520-0469(1971)028<0702:DOADOI>2.0.CO;2

[jgrd55919-bib-0016] Madden, R. A. , & Julian, P. R. (1972). Description of global‐scale circulation cells in the tropics with a 40–50 day period. Journal of the Atmospheric Sciences, 29(6), 1109–1123. 10.1175/1520-0469(1972)029<1109:DOGSCC>2.0.CO;2

[jgrd55919-bib-0017] Marshall, A. G. , Hendon, H. H. , Son, S.‐W. , & Lim, Y. (2017). Impact of the quasi‐biennial oscillation on predictability of the Madden‐Julian oscillation. Climate Dynamics, 49(4), 1365–1377. 10.1007/s00382-016-3392-0

[jgrd55919-bib-0018] Martin, Z. , Wang, S. , Nie, J. , & Sobel, A. (2019). The influence of the quasi‐biennial oscillation on the Madden‐Julian oscillation in idealized cloud‐resolving simulations. Journal of Geophysical Research: Atmospheres, 76, 669–688.

[jgrd55919-bib-0019] Neale, R. B. , et al. (2012). Description of the NCAR Community Atmosphere Model (CAM 5.0), NCAR Tech. Note NCAR/TN‐486 + STR, 268 pp., Natl. Cent. for Atmos. Res., Boulder, Colo. Retrieved from http://www.cesm.ucar.edu/models/cesm1.0/cam/

[jgrd55919-bib-0020] Nie, J. , & Sobel, A. H. (2015). Responses of tropical deep convection to the QBO: Cloud resolving simulations. Journal of the Atmospheric Sciences, 72(9), 3625–3638. 10.1175/JAS-D-15-0035.1

[jgrd55919-bib-0021] Nishimoto, E. , & Yoden, S. (2017). Influence of the stratospheric quasi‐biennial oscillation on the Madden–Julian Oscillation during austral summer. Journal of the Atmospheric Sciences, 74(4), 1105–1125. 10.1175/JAS-D-16-0205.1

[jgrd55919-bib-0022] Pegion, K. , Kirtman, B. P. , Becker, E. , Collins, D. C. , LaJoie, E. , Burgman, R. , Bell, R. , DelSole, T. , Min, D. , Zhu, Y. , Li, W. , Sinsky, E. , Guan, H. , Gottschalck, J. , Metzger, E. J. , Barton, N. P. , Achuthavarier, D. , Marshak, J. , Koster, R. D. , Lin, H. , Gagnon, N. , Bell, M. , Tippett, M. K. , Robertson, A. W. , Sun, S. , Benjamin, S. G. , Green, B. W. , Bleck, R. , & Kim, H. (2019). The Subseasonal Experiment (SubX): A multi‐model subseasonal prediction experiment. Bulletin of the American Meteorological Society, 100(10), 2043–2060. 10.1175/BAMS-D-18-0270.1

[jgrd55919-bib-0023] Richter, J. H. , Solomon, A. , & Bacmeister, J. T. (2014a). Effects of vertical resolution and nonorographic gravity wave drag on the simulated climate in the Community Atmosphere Model, version 5. Journal of Advances in Modeling Earth Systems, 6(2), 357–383. 10.1002/2013MS000303

[jgrd55919-bib-0024] Richter, J. H. , Solomon, A. , & Bacmeister, J. T. (2014b). On the simulation of the quasi‐biennial oscillation in the Community Atmosphere Model, version 5. Journal of Geophysical Research: Atmospheres, 119(6), 3045–3062. 10.1002/2013JD021122

[jgrd55919-bib-0026] Son, S.‐W. , Lim, Y. , Yoo, C. , Hendon, H. H. , & Kim, J. (2017). Stratospheric control of the Madden–Julian Oscillation. Journal of Climate, 30(6), 1909–1922. 10.1175/JCLI-D-16-0620.1

[jgrd55919-bib-0027] Stan, C. , Straus, D. M. , Frederiksen, J. S. , Lin, H. , Maloney, E. D. , & Schumacher, C. (2017). Review of tropical‐extratropical teleconnections on intraseasonal time scales. Reviews of Geophysics, 55(4), 902–937. 10.1002/2016RG000538

[jgrd55919-bib-0028] Vitart, F. (2017). Madden—Julian Oscillation prediction and teleconnections in the S2S database. Quarterly Journal of the Royal Meteorological Society, 143(706), 2210–2220. 10.1002/qj.3079

[jgrd55919-bib-0029] Vitart, F. , Ardilouze, C. , Bonet, A. , Brookshaw, A. , Chen, M. , Codorean, C. , Déqué, M. , Ferranti, L. , Fucile, E. , Fuentes, M. , Hendon, H. , Hodgson, J. , Kang, H. S. , Kumar, A. , Lin, H. , Liu, G. , Liu, X. , Malguzzi, P. , Mallas, I. , Manoussakis, M. , Mastrangelo, D. , MacLachlan, C. , McLean, P. , Minami, A. , Mladek, R. , Nakazawa, T. , Najm, S. , Nie, Y. , Rixen, M. , Robertson, A. W. , Ruti, P. , Sun, C. , Takaya, Y. , Tolstykh, M. , Venuti, F. , Waliser, D. , Woolnough, S. , Wu, T. , Won, D. J. , Xiao, H. , Zaripov, R. , & Zhang, L. (2017). The Sub‐seasonal to Seasonal (S2S) Prediction Project Database. Bulletin of the American Meteorological Society, 98(1), 163–173. 10.1175/BAMS-D-16-0017.1

[jgrd55919-bib-0030] Wang, S. , Tippett, M. , Sobel, A. H. , Martin, Z. , & Vitart, F. (2019). Impact of the QBO on prediction and predictability of the MJO convection. Journal of Geophysical Research: Atmospheres, 124 10.1029/2019JD030575

[jgrd55919-bib-0031] Wheeler, M. C. , & Hendon, H. H. (2004). An all‐season real‐time multivariate MJO index: Development of an index for monitoring and prediction. Monthly Weather Review, 132(8), 1917–1932. 10.1175/1520-0493(2004)132<1917:AARMMI>2.0.CO;2

[jgrd55919-bib-0032] Yoo, C. , & Son, S.‐W. (2016). Modulation of the boreal wintertime Madden‐Julian Oscillation by the stratospheric quasi‐biennial oscillation. Geophysical Research Letters, 43(3), 1392–1398. 10.1002/2016GL067762

[jgrd55919-bib-0033] Zhang, C. , & Zhang, B. (2018). QBO‐MJO connection. Journal of Geophysical Research: Atmospheres, 123(6), 2957–2967. 10.1002/2017JD028171

